# Supplementary material for: Developmental and genetic modulation of evidence integration dynamics in zebrafish sensorimotor decision-making
Source: bioRxiv. 2026 Mar 3:2026.03.01.708829. Preprint. [Version 1] doi: 10.64898/2026.03.01.708829 (PMC12991127; doi:10.64898/2026.03.01.708829)
Supplement: Supplement 4 [file NIHPP2026.03.01.708829v1-supplement-4.pdf]

bioRxiv preprint doi: https://doi.org/10.64898/2026.03.01.708829; this version posted March 3, 2026. The copyright holder for this preprint (which was not certified by peer review) is the author/funder, who has granted bioRxiv a license to display the preprint in perpetuity. It is made available under aCC-BY 4.0 International license.

SUPPLEMENTARY MATERIAL

Developmental and genetic modulation of evidence integration dynamics in zebrafish sensorimotor decision-making

AUTHORS

Roberto Garza[1,2,4,*], Ahmed El Hady[2,4,‡], Armin Bahl[1,2,3,4,‡,*]

AFFILIATIONS

[1] Department of Biology, University of Konstanz

[2] Centre for the Advanced Study of Collective Behaviour, University of Konstanz

[3] Zukunftskolleg, University of Konstanz

[4] International Max Planck Research School for Quantitative Behaviour, Ecology and Evolution (IMPRS/QBEE); Max Planck Institute of Animal Behavior, Konstanz, Germany

[‡] These authors jointly supervised this work

* Correspondence to roberto.garza@uni-konstanz.de; armin.bahl@uni-konstanz.de

Extended Data Figs. 1–4.

bioRxiv preprint doi: https://doi.org/10.64898/2026.03.01.708829; this version posted March 3, 2026. The copyright holder for this preprint (which was not certified by peer review) is the author/funder, who has granted bioRxiv a license to display the preprint in perpetuity. It is made available under aCC-BY 4.0 International license.

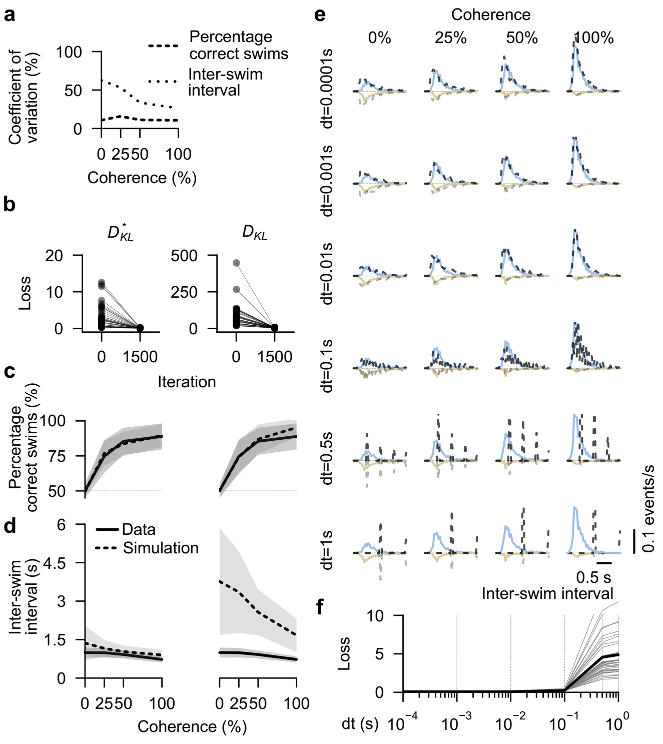

Extended Data Fig. 1 | Quantification of behavioral variability. Optimization results with two different loss functions and simulation time steps. a, Coefficients of variation of percentage correct (long dashed) and inter-swim interval (shorter dashes) for different coherence levels of larvae from **Fig. 1g**. **b**, Loss function reduction before and after fit of experimental data from N=39 5 dpf real larvae, using the improved (D^*_{KL}) and standard (D_{KL}) forms of the Kullback-Leibler divergence (**Methods**). Despite their difference in initial value, both loss functions converge to near-zero. **c,d**, Percentage of correct swims and inter-swim intervals plotted against the coherence level for the experimental (solid line) and fitted model (dashed line). The shaded areas surrounding each line represent the respective standard deviations across fish and fitted models. Optimization using D^*_{KL} leads to better matches of the target experimental data (left side) compared to results obtained using D_{KL} (right side). **e**, Model fits using different simulation time resolutions dt (labeled on the left side of each row). The target dataset was always the same experimental real fish. **f**, Final loss as a function of dt for all N=39 5 dpf real fish. Thin gray lines indicate single fish; the thick black line is their average. The final loss stably remains near zero for $dt \leq 0.01s$. All fish are the same individuals as in **Fig. 1g**. Related to **Figs. 1** and **2.**

bioRxiv preprint doi: https://doi.org/10.64898/2026.03.01.708829; this version posted March 3, 2026. The copyright holder for this preprint (which was not certified by peer review) is the author/funder, who has granted bioRxiv a license to display the preprint in perpetuity. It is made available under aCC-BY 4.0 International license.

Extended Data Fig. 2 | Bayesian optimization can extract latent model parameters from a broad range of datasets using experimentally feasible time scales. a, Parameter sets of 100 randomly generated models. These models fulfill a list of criteria to make them biologically realistic, but are not fit to experimental data (**Methods**). Gray lines connect values of the parameters belonging to the same model. **b,** Left: Evolution of loss and parameter estimation error over optimization iterations. Colored thin lines represent individual model fits. The black line shows the median across models. Right: Distribution of values at the beginning of (light colors) and after (dark colors) optimization. **c,** Loss and absolute parameter estimation errors obtained for datasets of different simulation lengths. For each length, we used the same 100 randomly generated models as in (a). The black line represents the average value across models. The black shaded area represents the corresponding standard deviation. As a control, in gray, the same results are shown for randomly initialized models before optimization. Asterisks (*) indicate significant differences ($p < 0.05$), comparing randomly initialized values to optimized ones. Double crosses (‡) display significant changes ($p < 0.05$), comparing optimized results from one simulation length to the next. Bootstrapping hypothesis testing was used in both cases. The arrow points to the size of experimental datasets. Related to **Fig. 2**.

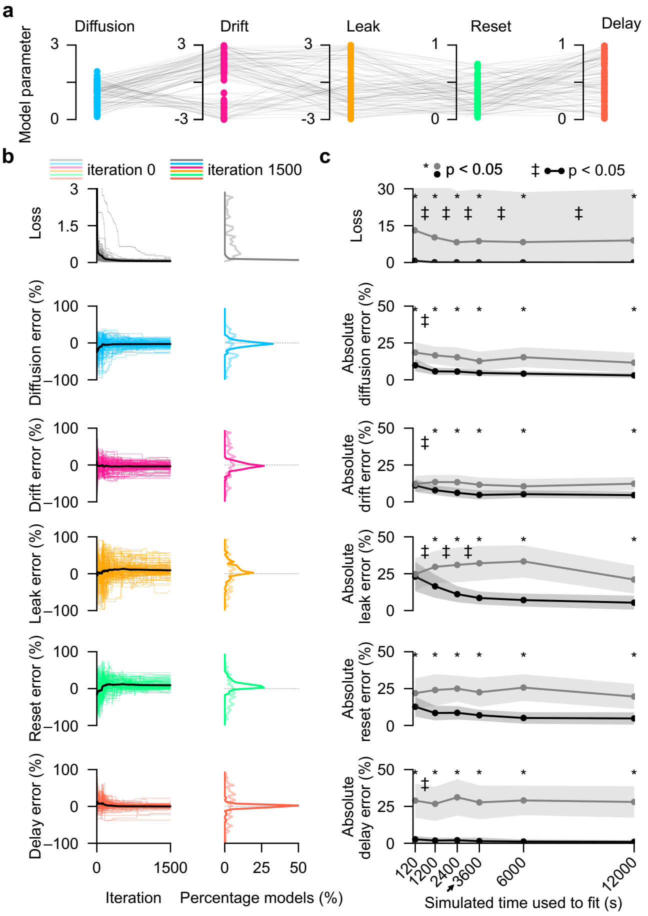

bioRxiv preprint doi: https://doi.org/10.64898/2026.03.01.708829; this version posted March 3, 2026. The copyright holder for this preprint (which was not certified by peer review) is the author/funder, who has granted bioRxiv a license to display the preprint in perpetuity. It is made available under aCC-BY 4.0 International license.

Extended Data Fig. 3 | Bayesian optimization is robust to noise and generalizes across experimental conditions. a, Distributions of inter-swim intervals for correct swims (blue) and incorrect swims (gold) at 50% coherence from an example simulated model. We added different amounts of Gaussian noise to the distributions. σ^2_{noise} indicates the noise variance. **b–g**, Distribution of loss function value and parameter estimation errors after optimization for each noise level for 100 randomly generated models. Same models as in **Extended Data Fig. 2a**. **h**, Average loss function value after optimization for simulations with added Gaussian noise, quantified from (b), as well as for various experimental conditions tested in the paper. Error bars represent standard deviation. **i,** Change of loss for small parameter perturbations after fitting models to experimental data from N=39 5 dpf real larvae (same individuals as in **Fig. 1g**). Perturbations are measured in percentage of the width of each parameter's search space. Thin colored lines link individual loss estimations (colored circles) of specific perturbation percentages for each fish and parameter. Related to **Figs. 2–5**.

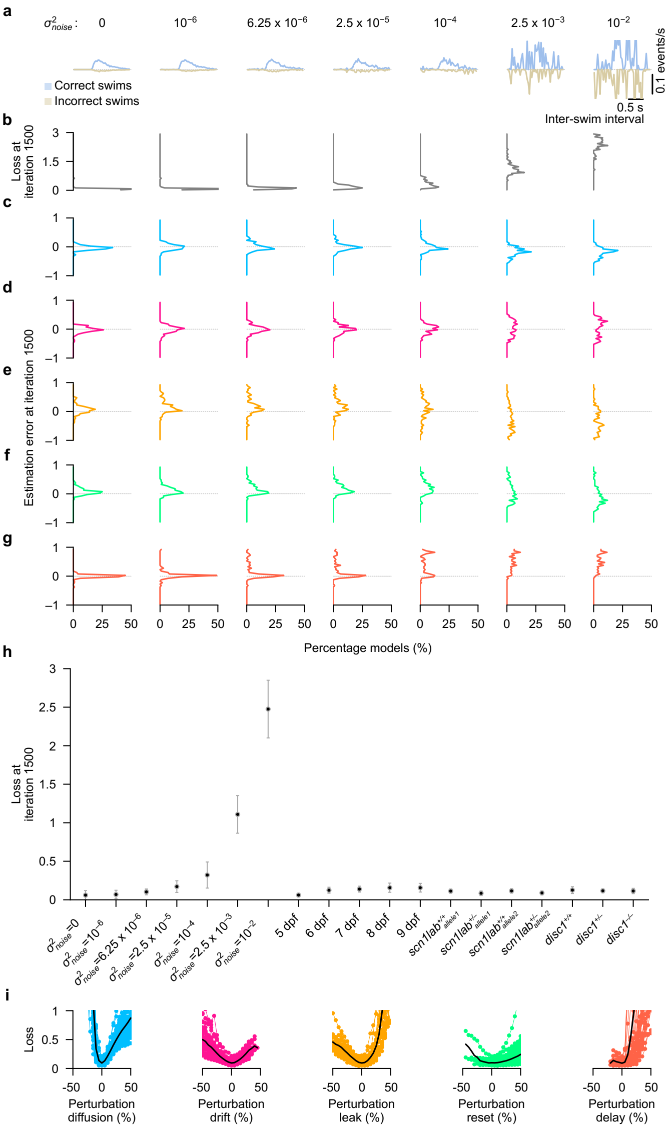

bioRxiv preprint doi: https://doi.org/10.64898/2026.03.01.708829; this version posted March 3, 2026. The copyright holder for this preprint (which was not certified by peer review) is the author/funder, who has granted bioRxiv a license to display the preprint in perpetuity. It is made available under aCC-BY 4.0 International license.

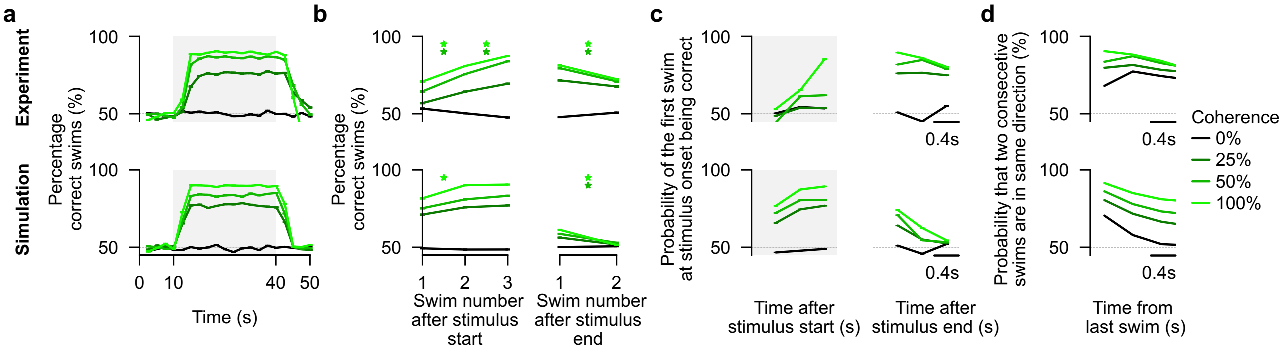

Extended Data Fig. 4 | Fitted drift-diffusion models can predict behavioral features not explicitly used during model generation. a, Percentage of correct swims, binned over time (with bins spanning 2.5 s) for all tested coherence levels. Gray boxes indicate the period of motion coherence display. Before and after, we display 0% coherence. The slow rise and decay of the curve at the start and stop of stimulus presentation indicate temporal integration and persistent motion memory. **b**, Percentage of correct swims increasing in the first three swims after the start of the stimulus (left) and decreasing in the first two swims after the end of the stimulus (right). Asterisks (*) indicate significant ($p < 0.05$, bootstrapping hypothesis test) changes in performance from one swimming event to the next at a given coherence level (different green levels). **c**, Probability of the first swim after the stimulus start (left) and after the stimulus end (right) to be correct. Swimming events were binned depending on the delay relative to the stimulus start (left) or end (right). **d,** Probability to consecutively swim in the same direction as a function of inter-swim interval during periods of 0% coherence. The slow decay indicates the tendency to consecutively repeat the same sensorimotor decision. The top row in (a–d) represents results from experimental data, merged from all animals. The bottom row in (a–d) shows model-generated results, merged from individual model simulations. N=39 fish and N=39 individual fish-fitted models, respectively. All experimental animals were 5 dpf old. Same animals and optimized models as in related **Fig. 3**.
